# Neurasthenia

**Published:** 1887-02

**Authors:** J. Edward Green

**Affiliations:** Macon, Ga.


### NEURASTHENIA.*

J. EDWARD GREEN, M. D., MACON, GA.

---

Neurasthenia is an undue exaltation of the whole nervous system, characterized by a general hyperæsthesia and irregular action of all the organs of the body.

ETIOLOGY.—Why is it we hear so much now-a-days of nervous prostration and debility and sudden and complete break-down

*Read before Macon Medical Society.

among all classes of society? Why is it that the merchant has so frequently to throw up his business, the professional man his clients and the student his studies ? The universal cry in every direction is nervous exhaustion, and to meet the wants of this new complaint, the country and the medical profession in every direction is deluged with nerve food, brain food, aids to digestion, *et hoc genus omne.* America is a new country, and among other things has brought into existence a great many new diseases. In the furious rush and excitement with which men carry on both business and pleasure, especially observable in the large cities of our country, may be seen the principal cause of the malady under consideration. Foreigners gaze with astonishment at the frenzied energy with which our people put forth every effort in pursuit of the almighty dollar. While contemplating the frantic struggle one is frequently prompted to pause and ask the question, why should so much be expended to procure such a small return ? For after all the nervous energy is gone, the capacity for enjoyment is likewise exhausted, and money affords but little compensation in proportion to the expenditure. Take for example the Wall street broker, who rises from an uneasy couch at 6 o'clock, or earlier in the morning, and after a hurried and unrelished meal dashes down to the stock exchange, where generally his whole mental faculties are upon a most intense strain till about six in the afternoon, when he returns to his home and hurriedly bolts a heavy meal, which might properly to be eaten with slowness and deliberation. After this, instead of throwing aside the cares and harass of the day, his ever-active brain goes on to form new plots and schemes and combinations for the morrow, and so the night is spent; instead of sleep coming to restore the havoc made by the untiring labors of the day, his brain cogitates on the fluctuations of the money market and the probable success or failure of his numerous ventures. At last this over-taxation of the nervous system begins to tell and insulted nature asserts herself. Soon such an individual begins to complain of insomnia and resorts to the pernicious practice of giving himself a sleeping draught of bromide or morphine; this sustains him for a variable period, after which it loses its effect and his symptoms return in an increased degree, and finally the machine gives way altogether and the nervous system is permanently debilitated and broken down, thus frequently necessitating an entire stoppage from business, or worse still, the brain itself gives way and general paralysis or cerebral softening is the result. The same mad whirl is characteristic of our institutions of learning; a young man, frequently without any preliminary preparation whatever, jumps from the plow to a medical or literary college, where he finds under such circumstances that he must necessarily compress the work of six years into two. The exertion requisite to such a task must of necessity be herculean. The privation, bad food, when they are frequently herded together in slums, exposure in all weathers, together with frequent dissipation indulged in with a sort of mad excess, all contribute their share to the final collapse, and then the last two or three weeks which precede the *final;* upon what an intense mental strain he is during this time; the mental functions are stretched to their uttermost capacity and every device is brought into play to keep the flagging energies up to the mark; iced bandages around the head and hypodermics of ammonia, brandy and morphine goad on the jaded intellects to the last. Such superhuman efforts necessarily leave a permanent impress upon the nervous system and at the same time render the organism much less able to withstand the invasion of disease. I know of five different cases who all succumbed at this period to the several effects of acute inflammatory rheumatism, small-pox, pneumonia and meningitis. The system of cramming, so much carried on in the public schools of the country, is greatly to be deprecated. A short time back the death of a girl of ten years of age was reported in Boston, with no assignable cause but over-application ; children whose minds are thus so unnaturally forced have, of course, but a very ephemeral existence.

This high-pressure mode of life creates inevitably an universal demand for stimulation, which is another great etiological factor in the production of the neurasthenic condition, and certainly aggravates instead of repairing the damage. Everything must be stimulated, for the business man must have his dram to get through the work of the day and the fine lady her matutinal draught to sustain her through the fatigues of society. This is followed by chloral tablets and ether to secure an artificial repose. Unnatural and early stimulation of the emotions and the cerebral functions soon brings about neurasthenia in females. Education also plays a considerable *role* in the development of this disease. The prolonged contact of children with older persons will develop in them a precocious intelligence and want of simplicity; the intellectual faculties of young girls are artificially stimulated at the expense of their physical powers. Their sensibility becomes excessively developed and their imagination excited by stimulating fiction, balls and plays. Feelings are thus aroused which are seriously at variance with the stern necessities of life. All these conditions, which are so often found united, especially in large cities, and which are frequently accompanied by anæmia and chlorosis, diminish the energy of the nervous system at an early period, stimulate the brain to an excessive degree and exaggerate the reflex excitability of the spinal cord. With respect to the manner of taking stimulants, the same element of rapidity is illustrated; a man will rush into a bar-room, gulp down a gill of raw whiskey and then bolt after it a swallow of ice-water, and out he goes, instead of sitting down quietly and sipping it with some solid food. Pretty soon dyspepsia and after a while Bright's disease sets in.

The immoderate use of coffee and tobacco must also be set down as among the many causes of this complaint. Coffee and alcohol combined, as some are in the habit of using them together, form a positive poison to the nerve centers. Among the physical causes may be mentioned spiritualism, mesmerism and theosophy, which at different times have taken such a hold on the public mind. It will be interesting to note just here that in the evolution of our peculiar civilization, the marvelous lengths to which the use of electricity has been pushed. In conformance with the morbid activity of the nervous system peculiar to America, our discoveries in this department actually border on the supernatural. The magnetic telegraph, the telephone, telegraphing on moving trains, and more wonderful than all the rest, the phonograph, the inventor of which a hundred years ago would certainly have been burned at the stake for commerce with the powers of darkness. Think of sending, for instance, two messages in opposite directions over the same wire, or several at the same time, and yet such feats have been accomplished so many times that they no longer excite our astonishment. With what superb traveling accommodations is America provided—wonders undreampt of even 50 years ago! The rolling palaces, fitted up with every superfluity of luxury, now make a journey a royal progress, which was only a few years back a "*pline forte et dure*." These gorgeous saloons, glittering with plate-glass and bronze, evenly heated with warm air, all parts brought in direct communication by means of electric bells, all show the desire for luxury that always accompanies an over-developed nervous system. Religious excitement also plays an important part in its production. Even in the modicum of time spent in reading may be seen the universal demand for stimulation. Books, the unhealthy productions of the diseased imaginations of such writers as Zola, Balzac and Collins, supply the necessary excitation for the hyperæsthetic brain. Lastly, the entire surrender to the mightiest and most irresistible of all the passions completes the work of destruction.

SYMPTOMS.—The neurasthetic individual is constantly in a state of pain; he is painfully conscious of the performance of all his functions—of his digestive tract, of his heart's action, of his lungs, and more than all the rest, of his brain. There is no period at which he is in a state of complete quiescence. These patients can frequently trace the progress of food or medicine through the whole alimentary canal, and note the exact period of time which it remains in each separate portion. This hyperæsthesia of the sympathetic system is pathognomonic and never absent. It is absolutely essential that the nerve-cells, emptied of their stored-up force during the day, must be replenished during the night if the organism is expected to act in a healthy and satisfactory manner; however, insomnia, which always attends this class of cases, increases the evil state of things brought about by other causes. One singular symptom is the frequent and irresistible impulse on the part of the neurasthenic to set in action one set of muscles, such for instance as to flex the forearm upon the arm, to clench the fist tightly, or in fact to contract, as far as possible, all the muscles of the body, this general muscular contraction resembling a mild tonic spasm, generally ends in the disagreeable sensation in the abdomen already referred to. These contractions and relaxations are caused very probably by certain irregular explosions of nervous force emanating from the motor ganglia of the cord. Why the nervous impulse should find an ultimate expression at the centre of the abdomen, I am at a loss to determine. Migraine and dyspepsia are almost constant symptoms, especially with that class of patients engaged in brain-work; the dyspepsia is sometimes reflex, sometimes of peripheral origin. Palpitations and irregular pulse are also frequently noticed. The bowels are sometimes loose and sometimes constipated, and the urine varies both in quality and quantity—in fact, irregularities in everything is the universal symptom of the disease. The sensory nerves express their irritability by vague neuralgias in every part of the body which are metastatic in their character, and the cutaneous nerves by constant itchings without any assignable cause. Owing to this constant contraction and relaxation of all the tissues, varying degrees of temperature prevail in different parts of the surface of the body. One hand or foot will be frequently uncomfortably hot, while its fellow will be as disagreeably cold. In addition to these variations I have observed a dry, stiff condition of the hair and skin, which is simply another result of defective cutaneous enervation, diminishing the amount of moisture which should come to the surface. In connection with the hair along the tract of a cranial nerve a gray streak is frequently caused by the proper pigmentary deposit being cut off when the nerves suffered from neuralgia. These subjects are almost always anæmic; however, the brain is sometimes hyperæmic and sometimes the reverse; thus excessive wakefulness and drowsiness coming on during the day alternate with each other. As to the eyes, asthenopia, amblyopia, photophobia and lachrymation are generally complained of. Sometimes the patient's sensitiveness to flashes of light is painfully exalted.

It is to be deplored, in this condition of things, the universal resort to stimulants, which inevitably comes sooner or later, and also the mode in which they are used. This disastrous mode of taking liquors into the system, before alluded to, only makes bad worse and precipitates the ultimate break-down. Alcohol thus coming in immediate contact with the stomach acts in two ways: it first brings on gastritis or chronic dyspepsia; and secondly, burns up more rapidly still the nervous energy already being destroyed by the wear and tear of business. As it is to be expected from the nature of the affection, a certain degree of inco-ordination is found most often in the muscles of the arm; frequently the first sign a patient will betray of his condition will be a marked change in the handwriting. To a close observer a slight change in the gait is likewise detected. In exploring along the spine and exerting pressure, in a great many cases the "*puncta dolorosa*" will be brought to the notice of both patient and practitioner. These tender spots are frequently not perceived till their existence is demonstrated by actual pressure.

With regard to the psychical functions, along with the other variations from the normal, there is a marked and profound disturbance; the neurasthenic is inclined to be peevish, irritable and fault-finding, and trifles which would be passed over unnoticed by a healthy individual throw them almost into a spasm; the power of inhibition seems to be almost entirely withdrawn, and such patients frequently commit themselves in such a manner as causes them afterwards great mental suffering. They enjoy in an inordinate degree, and likewise suffer too keenly; in fact, they feel too much; their pleasures and pains are out of all proportion to the causes from which they arise. Too much sensation, both mental and physical, is compressed within too short a period; the mighty rush of emotions of all sorts is to them frequently appalling. Thus the capacity for emotion being gradually exhausted by the unceasing fret and worry of the minute details of life, when an occasion does arise where it ought properly to be called forth, the neurasthenic is scarcely able to express it. The *morale* is to a great extent subverted; a person so affected is so largely occupied by his own aches, and pains, and symptoms that all sense of obligation to others is dwarfed into insignificance.

The extreme degree of hyperæsthesia of the auditory nerves has already been alluded to; noises of all sorts, a simple rapping with the hand on the table, the rumbling of carriages and the ringing of bells frequently produce positive torture. This over-sensitiveness to sounds in its proper place is simply the musical sense perverted. It is observable oftentimes that a family of neurotic tendency produces a child in which the musical faculty is inordinately developed; all the nervous force is merged in this one instinct. Only those who are musicians themselves can completely comprehend how slight a cause suffices to disturb that exquisite equipoise necessary to the perfect rendering of any composition. There is nothing in the whole range of manual dexterity that requires the nervous system to be more *in tune*, so to speak, than manipulation of the piano keys. The efferent nerves must convey with absolute precision the reflex impulse coming from the registry in the cord. The feverish erethism of a pianist appearing before his audience will be affected to such an extent by the trifling accident of tripping the foot, a slight catarrh, or a headache that to a practiced ear will vitiate the whole performance. On view ing a picture, I have heard many say they do not take in the whole scheme satisfactorily to themselves, and likewise with a written passage, the whole meaning is rarely grasped till after several readings. This uneven, irregular functioning of the cerebral faculties is often the sure precursor of some of the different forms of manias.

I should not omit to present to your consideration the appalling number of suicides, particularly among very young persons, daily filling our journals, as another manifestation of nervous debility.

The terminations of neurasthenia, if the cause be not removed in time, are numerous, Bright's disease, hemiplegia, cerebral softening, lacomotorataxia and general paralysis of the insane are only more complete expressions of general nervous exhaustion.

The morbid pathology of this multitudinous congeries of symptoms, recently considered as a separate disease, reveals very little; when it eventuates in general paralysis, as above stated, diffused patches of sclerosed tissue will be found scattered indifferently along the cord and the base of the brain. When

Bright's disease is the result, the lesions appropriate to that affection will be disclosed on examination of the kidney. Sometimes the one or both hemispheres of the brain are in a shrunken condition from partial or complete absorption of the neuroglia. In general paralysis, the cortex especially is in a state of atrophy. With regard to the blood, a tendency to leukosytosis is mostly noticed, the percentage of the white blood-cells being much larger than normally.

TREATMENT.—The *rationalé* of physic now being to remove the cause, that, then, is the first consideration; let the merchant retrench his business schemes, the student his application, and the man of pleasure his exciting dissipations. That element of rush and feverish restlessness so characteristic to American life it is most desirable to tone down. The important fact that a certain time should be set apart for pleasure and relaxation is not sufficiently attended to. Rest and sleep, then, are the two first indispensable conditions to the therapy of weak nerves. A change is always in order that rest and sleep may become possible. A simple removal from the harass and worry and carping anxiety of business, especially of a speculative character, frequently enables the nervous system to repair the ravages inflicted upon it. The diet must be generous, and the patient enjoined strictly to eat slowly, and to masticate thoroughly all that passes into his stomach. The stomach, however, sharing the general irritability of the rest of the organism, must not be over-crowded by a multiplicity of articles. For medicine I strongly advocate lager-beer to be taken with the dinner, and only then. The advantages of this beverage are three-fold, the bitter principle as a tonic, the mild stimulus and the general sedative effect of the lupulin. I have little or no faith in the innumerable preparations of nerve and brain food which now deluge the country. The idea with the manufacturers of these mixtures is that they can repair the waste of all nervous tissue by cramming the stomach with two or more elements necessary to its existence. The brain and the nerves cannot be directly repaired by phosphorus fed into the stomach either in large or small quantities, but it is the whole economy that is suffering from malnutrition. Phosphorus, strychnine and electricity, so much vaunted as specifics in this affection, I consider as positive irritants; they are all stimulants of the most violent sort, when the patient is suffering from the effects of excessive stimulation of all sorts, when a little more stimulation would goad on the jaded energies to the utmost stretch of their capacity and certainly finish him off. In extremely anæmic subjects, the ferruginous preparations may be used with benefit. But of external adjuvants, surf-bathing and massage are *par excellence* the best; the action of salt water upon the skin has a more magical effect in restoring depressed energy than anything I know of. Lastly I would say, instead of cramming the stomach with all sorts of tonics and bromides and so-called brain and nerve food, give a fair and reasonable trial to the *Vis medicatrice naturæ.*

---

SCOPOLINE.—This new mydriatic, introduced by Pierd'houy, is, as far as my experience of it goes, a useful drug. Since the beginning of the current year I have employed it almost continuously amongst my out-patients at the West London Hospital, not for the purpose of testing its qualities as a mydriatic, but as a drug to supersede atropine in the treatment of keratitis, corneal ulcers and iritis. I have found that in the case of troublesome corneal ulcers which had been treated respectively with both atropine and eserine without success, rapid improvement followed the installation of scopoline; and especially was this good effect shown in cases of severe interstitial keratitis, in which atropine had been previously employed. Again, in rheumatic iritis, the use of scopoline was obviously effective in reducing the pain and injection of the globe. Upon no occasion have I seen any conjunctival or other irritation set up by its use, and I have found one grain to the ounce a sufficiently strong solution for my purposes. I am disposed to believe that, in addition to its mydriatic power, scopoline is able to effect some control upon the vascular supply of the eye; the drug may be indeed the physiological antithesis of eserine; at present I am engaged upon an inquiry, with Dr. P. S. Abraham, into the physiology and chemistry of the drug, and we hope before long to publish the results. H. PERCY DUNN, F. R. C. S.—*British Medical Journal*, Jan. 8th, 1887.

